# Cell‐Free DNA as a Biomarker of Cell Death and Treatment Response for On‐Chip Three‐Dimensional Tumor Models

**DOI:** 10.1002/adhm.71792

**Published:** 2026-10-04

**Authors:** Maryam Ziaee, Julie Lafontaine, Badr Id Said, Kousha Kamal, Kim Tsoi, Francis Rodier, Thomas Gervais, Philip Wong

**Affiliations:** ^1^ Institute of Biomedical Engineering Polytechnique Montréal Montréal Quebec Canada; ^2^ Centre de Recherche du Centre Hospitalier de L'université de Montréal (CRCHUM) Montréal Quebec Canada; ^3^ Institut du Cancer de Montréal (ICM) Montréal Quebec Canada; ^4^ Radiation Medicine Program Princess Margaret Hospital Toronto Ontario Canada; ^5^ Department of Radiation Oncology University of Toronto Toronto Ontario Canada; ^6^ Biomaterials and Biomedical Engineering University of Toronto Toronto Ontario Canada; ^7^ Department of Orthopedic Surgery Mount Sinai Hospital Toronto Ontario Canada; ^8^ Department of Radiology, Radio‐Oncology and Nuclear Medicine Université de Montréal Montréal Quebec Canada; ^9^ Department of Engineering Physics Polytechnique Montréal Montréal Quebec Canada

**Keywords:** cell‐free DNA, micro‐dissected tumor explants, on‐chip 3D models, sarcoma, spheroids

## Abstract

On‐chip 3D cancer models are important tools to investigate treatment response and tumor biology. With their increasing adoption, there is a need for non‐destructive methods that enable the sequential monitoring of cell death and tumor response over time. This study presents quantifying cell‐free DNA (cfDNA) as a proxy for cell death for on‐chip 3D sarcoma models. qPCR analysis of media cfDNA from spheroids and micro‐dissected tumor explants (MDTs) generated from SK‐LMS‐1 and MCA‐205 cell lines cultured on microfluidic chips showed a correlation between baseline cfDNA concentration and on‐chip tumor burden. Furthermore, cfDNA levels increased in a dose‐dependent manner following cytotoxic treatments, including staurosporine and hydrogen peroxide. Single‐dose radiation therapy also raised cfDNA levels in media from MCA‐205 but not SK‐LMS‐1 MDTs, in accordance with their 2D culture response. Finally, cfDNA release within the first days of culture was shown to reflect patient‐derived MDT survival, addressing a critical unmet need for ex vivo culture. This study suggests that media cfDNA quantification could be used as a reliable, non‐disruptive, and rapidly quantifiable measure of cell death for on‐chip 3D models, which facilitates more robust use of 3D model systems for pre‐clinical assessment of treatment‐induced cell death and biological response.

## Graphical abstract

This study presents quantifying cell‐free DNA (cfDNA) as a non‐disruptive and rapidly quantifiable method for assessing cell death for on‐chip 3D sarcoma tumor models. On‐chip cfDNA release reflects tumor burden, extent of cell death, and survival of in vitro and ex vivo 3D models, providing valuable insight into the temporal response of tumor models to various treatments. *(Figure created using BioRender.com)*.

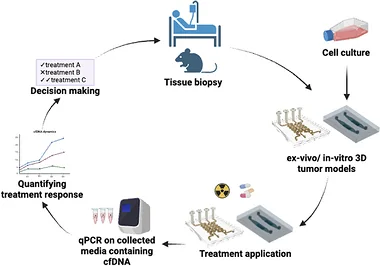

**Abbreviations**
BSA: Bovine serum albumin
CC3: Cleaved Caspase‐3
cfDNA: cell‐free DNA
CRCHUM: Centre Hospitalier de l'Université de Montréal
D‐PBS: Dulbecco's phosphate‐buffered saline
FBS: Fetal bovine serum
H&E: Hematoxylin and eosin
H_2_O_2_
: Hydrogen peroxide
IF: Immunofluorescent
LINE: Long interspersed nuclear element
MDT: Micro‐dissected tumor explants
PI: Propidium iodide
qPCR: Quantitative polymerase chain reaction
RT: Radiation therapy
STS: Soft tissue sarcoma

## 1 Introduction

Three‐dimensional (3D) tumor models are increasingly used to study cancer and its response to various treatments [1, 2, 3]. They are physiologically more representative of cancers than monolayer cell cultures and are less expensive, time‐consuming, and labor‐intensive than animal models [1, 2, 3]. Indeed, the FDA Modernization Act 2.0 and 3.0 allow and encourage researchers to use alternative models—including diverse 3D models—to replace animal surrogates for drug discovery purposes [4, 5]. 3D tumor models can range from simple spheroid culture to more complex models, such as tumor organoids and ex vivo tissue slices and micro‐dissected tissue explants (MDTs) derived from patient or xenografted samples [6, 7, 8, 9]. If cultured properly, the latter models can preserve the integrity of the tumor cellular and architectural components and have the potential to serve as patient‐specific models to predict patient treatment response [8, 10, 11].

Microfluidic chips can be used to maintain and manipulate 3D tumor models [12, 13]. Tumor‐on‐chip models confer the advantages of reduced reagent consumption, increased throughput, and precise control over experimental conditions compared to plate‐based models [12, 13, 14]. Moreover, microfluidic chips allow for enhanced viability of ex vivo models such as MDTs over long periods of time [15, 16]. However, a major bottleneck to their use stems from the lack of standardized, reliable, and non‐destructive measures for cell death and viability, as most commercial assays are developed for 2D models [17, 18].

Live cell imaging of 3D models, which is done through fluorescence microscopy, is limited by light scattering, and the signal is strongly surface‐weighted [19, 20, 21]. This could be improved by sophisticated microscopy techniques, such as two‐photon microscopy, but they are still low‐throughput and lack the spatial resolution to capture the depth of the 3D samples [22]. Furthermore, integrating biosensors within the microfluidic chip is not compatible with all culture devices [23]. Oftentimes, to assess the cytotoxicity of a treatment, destructive end‐point measurements are used, which include sample fixation or dissociation for biomarker staining using immunofluorescent/immunohistochemistry [11, 24, 25]. These techniques acquire a snapshot picture of the sample, and experimental outcomes are dependent on the precise timing and number of events captured at that single time point. Although using the effluent of the chip to assess cell viability and death by measuring the secretion of molecules like lactate dehydrogenase or ATP has been tested in various studies, these luminescence‐ or fluorescence‐based assays inherently have high background noise, which reduces their sensitivity [26, 27].

Cell‐free DNA (cfDNA) is the DNA fragment that is released from cells into the surrounding media through cell death and, to a lesser degree, active secretion [28, 29, 30]. cfDNA originating from tumor cells, referred to as circulating tumor DNA (ctDNA), is increasingly being tested as a means for cancer screening, diagnosis, patient prognostication, treatment response prediction, and post‐treatment monitoring [31, 32]. Drawing a parallel from their clinical applications, we hypothesize that cfDNA in the chip's media can be an alternative robust surrogate for cell viability and death in 3D models (Figure 1). In this study, we used soft tissue sarcoma (STS) as our cancer model. Using monolayer culture, spheroids, and MDTs from STS xenografts and patient biopsies, we investigated the correlation between tumor burden and cfDNA release as a baseline measure of viability. We then characterized the dynamics of cfDNA release from these models in response to different treatments, including staurosporine (primarily an apoptosis‐inducing agent) and Hydrogen Peroxide (primarily a necrosis‐inducing agent), as well as radiation therapy (RT), a more clinically relevant treatment with diverse effects on cell fate.

**FIGURE 1:**
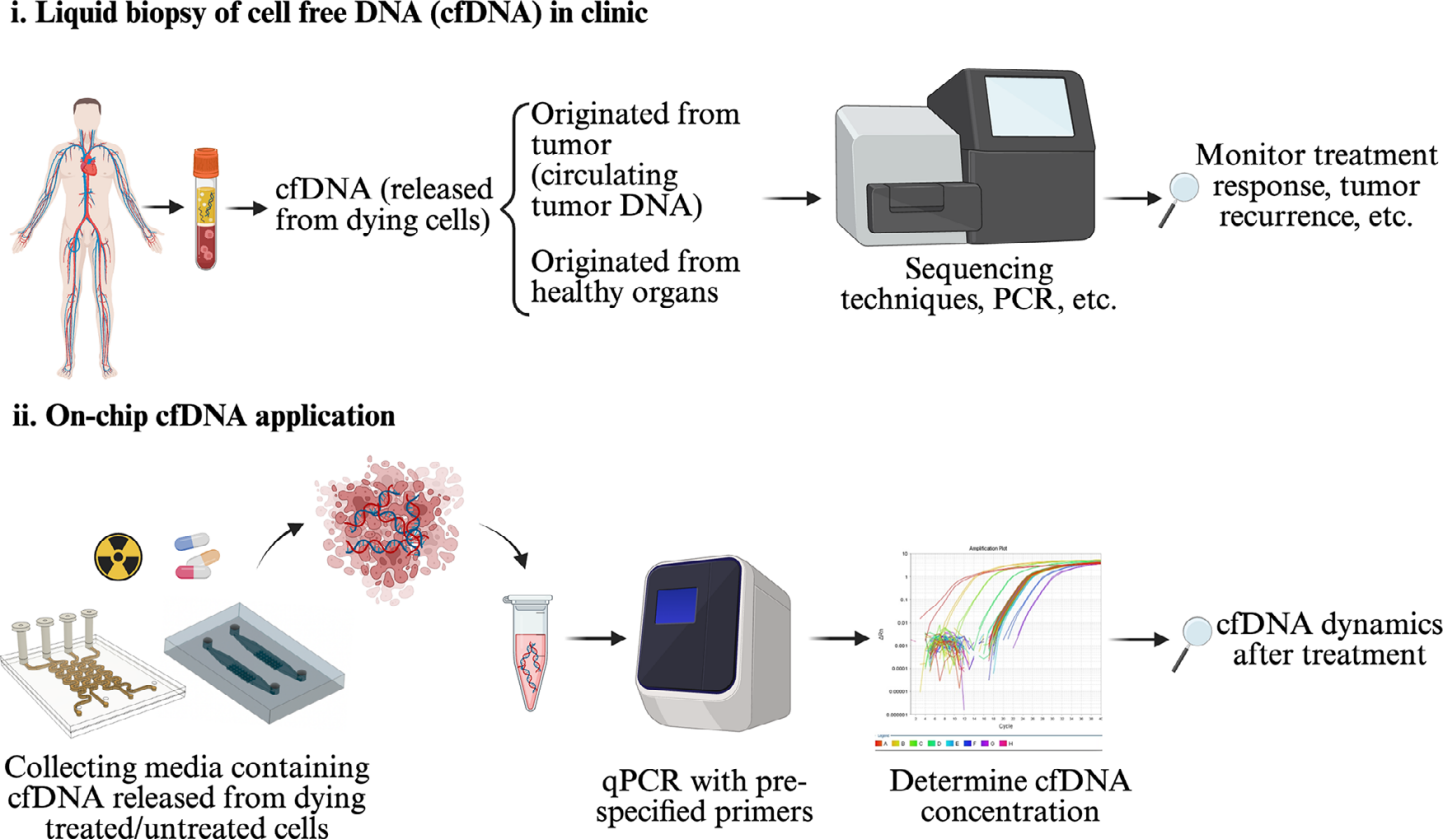
Graphical overview of the application of cfDNA quantification in liquid biopsy as well as for cell death measurement of on‐chip 3D tumor models. (i) Representation of the practical use of cfDNA in liquid biopsy technique for cancer patients. (ii) Our modification of the same method to quantify cell death pre/post treatment for on‐chip 3D tumor models. This figure was created with BioRender.com.

## 2 Methods

### 2.1 Microfluidic Device Fabrication and Preparation for Spheroid and MDT Culture

The characteristics of microfluidic chips used for spheroids and MDTs are depicted in Figure S1. The MDT chips, the design of which was previously established, were purchased from MISO Chip (Montreal, Quebec, Canada) [11]. They consisted of four channels each harboring eight 0.8 × 0.8 × 0.7 mm wells specifically designed to trap 0.5 mm tissue explants and culture them without perfusion. The design of spheroid chips was adopted from Patra et al., and comprised sedimentation wells into which 24 spheroids could be formed in a single pipetting step [33]. Briefly, the chip consisted of a top layer with two fluidic channels featuring an inlet and outlet of 3.2 mm diameter and a central rectangular area of 3.3 × 0.85 mm cross section and a bottom layer containing an array of 3 × 8 cubic wells with a width of 0.7 mm. The two layers were fabricated in Polydimethylsiloxane (PDMS) using soft lithography techniques on 3D‐printed molds and bonded together after exposure to atmospheric plasma, the procedure of which is found elsewhere [33].

Prior to 3D culture in corresponding chips, the channels were sterilized using an autoclave (for spheroid chips) as well as washed with Isopropanol or ethanol to remove air bubbles (for spheroid and MDT chips). The channels were then incubated for at least 24 h with PEG‐PPG‐PEG, Pluronic F‐108 (Sigma–Aldrich Canada Co., Oakville, ON, Canada) to prevent cell attachment to their surface. Just before the introduction of cells (for spheroid formation) or MDTs, the channels were primed with appropriate supplemented cell culture media or Hank's Balanced Saline Solution (HBSS, Wisent Inc., St‐Bruno, QC, Canada) supplemented with 1% penicillin–streptomycin solution (Wisent Inc.) and 0.6 mg/L amphotericin B (Wisent Inc.), respectively.

### 2.2 Cell Culture

SK‐LMS‐1 Human leiomyosarcoma cell line was procured in 2019 from the American Type Culture Collection (Cat#: HTB‐88, ATCC, Manassas, VA, USA). MCA‐205 murine fibrosarcoma cell line was kindly provided by Prof. Bertrand Routy (Cat#: SCC173, Montreal Cancer Institute, Montreal, QC, Canada) and was authenticated using short tandem repeat (STR) profiling in 2024. SK‐LMS‐1 and MCA‐205 cells were cultured in EMEM (Wisent Inc.) and RPMI 1640 1X with L‐Glutamine (Wisent Inc.), respectively, both supplemented with 10% fetal bovine serum (FBS) (Wisent Inc.) and 1% Penicillin–Streptomycin solution. For MDT culture, 0.6 mg/L amphotericin B was also added to the media.

### 2.3 2D Cell Culture in 96 Well Plates and Live Imaging of the Cells

SK‐LMS‐1 and MCA‐205 cells were seeded in 96‐well plates at 500–8000 cells/well. After 24 h, the media was changed to remove the unattached cells. On day 2, the media from the wells were collected, and the wells were supplemented with media containing Propidium Iodide (PI, 0.5 µg/mL, Sigma–Aldrich Canada Co.). After 1–2 h, wells were imaged with the IncuCyte S3 Live‐cell Imaging System (Sartorius, Göttingen, Germany) with 10× objective to look for confluency and PI^+^ cells (i.e., cells uptaking PI, which is a marker for cell death).

### 2.4 2D Cell Treatment With Staurosporine, Hydrogen Peroxide, and RT

Two days after seeding cells at 2000 density, cells were treated with staurosporine (APExBIO Technology, Houston, TX, USA) by supplementing the media with the desired final drug concentration (5 µM for SK‐LMS‐1 and 1 µM for MCA‐205). SK‐LMS‐1 cells were also treated with 5 mM Hydrogen Peroxide (H_2_O_2_). Some wells were supplemented with PI and Invitrogen CellEvent Caspase‐3/7 Green ReadyProbes Reagent (2 drops/mL, Invitrogen, ThermoFisher Scientific, Ontario, Canada) to monitor apoptosis and cell death over time using the IncuCyte System by measuring confluency, as well as green (Cleaved Caspase‐3^+^) and red (PI^+^) objects. Every 24 h after treatment, until 72 h, the media were collected and changed with fresh media. All the collected media were centrifuged for 5 min at 1500 rpm immediately after collection, diluted 1:10 in DNA buffer (Teknova, USA), and stored at −80°C until use for qPCR.

To determine the sensitivity of SK‐LMS‐1 and MCA‐205 cells to RT, the cells in suspension were irradiated at 8 Gy with a Gammacell 3000 irradiator (Best Theratronics, Ottawa, ON, Canada) and seeded at ∼4000 cells/well. Five days later, PI was added to the wells, and cells were imaged using the IncuCyte system.

### 2.5 Spheroid Cell Culture in Microfluidic Device

Cell seeding was conducted when cells reached around 80% confluency. We adopted a previously published method to form spheroids on‐chip [9]. Briefly, cells were detached from cell culture flasks using 0.25% trypsin‐EDTA (Wisent Inc.) for 2–3 min at 37°C, suspended in supplemented culture media, and centrifuged at 1500 rpm for 5 min. After that, the cell pellets were resuspended in a sufficient volume of culture media to get the desired densities (between 0.5–4 × 10^6^ cells/mL) for cell seeding in microfluidic devices. Then, 140 µL of cell suspension was pipetted three times into the inlet and three times into the outlet to ensure homogeneous cell seeding. The chips were then incubated at 37°C in a 5% CO_2_ incubator, and their media were changed every 24 h. At each media change interval, the chips’ media were collected and centrifuged for 5 min at 1500 rpm, diluted in DNA buffer (1:10, Teknova, USA), and stored at −80°C until use.

The spheroids were imaged every day using a Nikon TE300 inverted microscope (Nikon Instruments Inc., Melville, NY, USA). The brightfield images were analyzed using ImageJ (ImageJ, Fiji General Public License) software to measure the spheroids’ diameter.

### 2.6 Mouse Xenograft

After subculturing to reach ∼80% confluency, SK‐LMS‐1 (1 × 10^7^ cells/mL) and MCA‐205 (5 × 10^6^ cells/mL) cell suspensions were prepared in cold Dulbecco's Phosphate‐Buffered Saline (D‐PBS, Wisent Inc.) + Matrigel (Corning, USA) and in cold D‐PBS, respectively. Then, two flanks of Nod/Rag1 immunocompromised mice (from a local colony) for SK‐LMS‐1 and C57Bl/6 female immunocompetent mice (from Charles River Laboratories, Saint‐Constant, QC, Canada) for the MCA‐205 cell line were injected with 200 and 100 µL of cell suspension, respectively. The animals were kept at the animal facility of the CRCHUM according to the Guidelines for the Care and Use of Laboratory Animals of the CRCHUM and approved by our institutional committee on animal care (CIPA). The mouse tumor sizes did not exceed the maximal tumor size permitted by CIPA, that is, 1500 mm^3^. When reaching a volume of 150–500 mm^3^ (with one SK‐LMS‐1 tumor exceeding this range and reaching approximately 1200 mm^3^), the xenografts were harvested and used for MDT production.

### 2.7 MDT Formation and Culture From Xenografts

Previously published methods were used to produce MDTs [8, 11]. In summary, the tumor samples were chopped with a tissue chopper into 350 µm‐thick tissue slices, which were then punched into MDTs with a 500 µm‐diameter tissue punch. Finally, MDTs were collected and deposited into the channels’ inlet and flowed through the channel into the wells by aspirating the fluid from the outlet. Once all desired wells contained an MDT, the channels were rinsed three times with warm, appropriately supplemented culture medium. The chips were kept in a 5% CO_2_ incubator at 37°C, and their media were changed every 24 h. Like spheroids, at each media change interval, the chips’ media were collected, centrifuged, diluted in DNA buffer, and stored at −80°C until use.

### 2.8 MDT Culture From Patient‐Derived Tumor Samples

Fresh tumor specimens were collected from four sarcoma patients undergoing surgical resection without neoadjuvant therapy at Mount Sinai Hospital in Toronto, Canada. The histopathological information of the collected tumors is provided in Table S1. All patients provided written informed consent under institutional research ethics board (REB) protocols (01‐0138‐U). Upon resection, the tumor samples were placed in HBSS, supplemented with 10% FBS (Gibco, Thermo Fisher Scientific), and transported to the lab on ice. MDTs were then produced as explained in the previous section and cultured in MDT chips in supplemented RPMI media. Every 24–72 h, as indicated in the results section (timepoints with cfDNA data available), the chips’ media were changed, collected, and stored at −80°C until use.

### 2.9 On‐Chip Treatment With Staurosporine, Hydrogen Peroxide, and RT

Three days after culture, spheroids and MDTs were treated with staurosporine (0–20 µM for SK‐LMS‐1 and 0–5 µM for MCA‐205) and Hydrogen Peroxide (5 and 20 mM for SK‐LMS‐1). To do that, the chips’ media were changed three times with media containing the desired concentration of the drug. The 3D models were incubated for 24 h with the drug before the media were changed again. For conventional RT, the chips’ media were changed immediately before irradiation with a Gammacell irradiator at 8 Gy. SK‐LMS‐1 and MCA‐205 MDTs were irradiated on days 2 and 3, respectively.

### 2.10 cfDNA Analysis Using qPCR

cfDNA in the supernatant was quantified by quantitative polymerase chain reaction (qPCR) targeting long interspersed nuclear element (LINE) in the human [29] or mouse [34] genome. Table S2 summarizes the primers used for amplifying DNA fragments containing human or mouse LINE regions (generated by Invitrogen, ThermoFisher Scientific, Ontario, Canada). Media from patient‐derived MDTs also underwent DNA extraction using QIAamp Circulating Nucleic Acid Kit (55114, Qiagen, Germantown, MD, USA) upon thawing to increase the assay's sensitivity. Genomic DNA from Human (11691112001 for spheroids and cell line‐derived MDTs and D4642 for patient‐derived MDTs, MilliporeSigma, ON, Canada) and mouse (69239, MilliporeSigma) were used to build a standard curve of DNA content. A 10 µL mix made of PowerTrack SYBR Green Master Mix (ThermoFisher Scientific, Ontario, Canada), 50 µM forward and reverse primers, nuclease free water, and media samples in 10:1:1:8 ratio was prepared into MicroAmp Fast Optical 96‐Well Reaction Plate, 0.1 mL (ThermoFisher Scientific) and run in Applied Biosystems StepOne Real‐Time PCR System (for spheroids and cell line derived MDTs) or Bio‐Rad CFX96 Real‐Time System with a C1000 Touch Thermal Cycler (for patient‐derived MDTs). Representative amplification curves for standard genomic DNA and their corresponding standard curves are depicted in Figure S2.

### 2.11 Spheroid Fixation, OCT Embedding, and Immunofluorescence Staining

Four hours after staurosporine treatment, spheroids were fixed with Formalin 10% (Fisher Scientific Company, ON, Canada) for 60 min. Spheroids were then washed with D‐PBS, flushed out of the chips, and included in optical cutting compound OCT (Leica, Buffalo Grove, IL, USA). After at least an overnight incubation so that spheroids could sediment, the OCT blocks were flash‐frozen on dry ice and stored at −80°C.

The OCT blocks were sectioned into 4–5 µm slices using a Leica Cryostat (Leica, Buffalo Grove, IL, USA) and mounted on Superfrost Plus Microscope Slides (Fisher Scientific). Slides were incubated at room temperature for 1 h with permeabilization buffer (PBS 1×, 3% Bovine Serum Albumin (BSA), and 0.05% Triton X‐100) and then with permeabilization buffer containing rabbit anti‐Cleaved Caspase‐3 (anti‐CC3, 1:200) antibody (9661, Cell Signaling Technology, Danvers, MA, USA) overnight at 4°C. Sections were then washed with PBS1× and incubated for another hour with secondary antibody buffer (PBS 1×, 3% BSA) with AlexaFluor‐647 antibody (1:650) (A31573, Invitrogen, USA). Finally, slides were stained with DAPI and mounted with ProLong Gold Antifade Mountant (Invitrogen, ThermoFisher Scientific, Waltham, MA, USA). Slides were scanned with a Zeiss fluorescence microscope with Axio‐Vision 4.0 software (Carl Zeiss AG, Jena, Germany).

### 2.12 MDT Fixation, Paraffin Embedding, and Staining

MDTs were fixed and underwent paraffin embedding using a previously published protocol developed in our lab to produce paraffin blocks with a microarray of MDTs [11]. The paraffin blocks were then sectioned into 4–5 µm slices using a microtome and mounted on TOMO hydrophilic adhesion slides (Matsunami, Bellingham, WA, USA). Subsequently, BenchMark XT automated slide preparation system (Ventana DISCOVERY ULTRA, Ventana Medical System Inc., Tucson, AZ, USA) was used to perform immunofluorescence (IF) staining using mouse anti‐Ki‐67 antibody (1:500 dilution for SK‐LMS‐1 cell line, 9449, Cell Signaling Technology) or rabbit anti‐Ki‐67 antibody (1:200 dilution for MCA‐205 cell line, Cat# MA5‐14520, ThermoFisher) and AlexaFluor‐488 secondary antibody (1:250) (A31571, Invitrogen). VECTASHIELD Antifade Mounting Medium with DAPI (H‐1200‐10, Vector Laboratories, Inc., Newark, CA, USA) was used for mounting the slides. Sections were then scanned with an Olympus fluorescence microscope (bx61vs, Olympus, Toronto, ON, Canada).

Hematoxylin and eosin (H&E) staining of patient MDTs was conducted at CAMiLoD facility at the University of Toronto. The slides were imaged by a Zeiss Axio Scan.Z1 microscope.

### 2.13 Immunofluorescence and H&E Image Analysis

All images were analyzed on VisiomorphDP software (VisioPharm, Hørsholm, Denmark). To calculate the percentage of CC3 level in IF images, the total area of the tissue stained with CC3 was divided by the total area of the tissue (measured using DAPI channel by setting a low threshold to identify the whole tissue). To calculate the nucleated area in H&E images, the hematoxylin area was divided by the total area of the tissue.

### 2.14 Statistical Analysis

All statistical analyses and graph designs were done using GraphPad Prism (Version 9.0.1, GraphPad, San Diego, CA, USA). The normality of all datasets was assessed using the Shapiro–Wilk test. For column significance analysis between two conditions, a student's *t*‐test was carried out. For column significance analysis between multiple conditions, ordinary one‐way ANOVA with Tukey's multiple comparisons test or Kruskal–Wallis with post hoc Dunn's test was performed for datasets with normal or non‐normal distribution, respectively. Comparisons between group measures at multiple time points were done using ordinary two‐way ANOVA with Šídák's multiple comparisons test. The correlations between cell number in 2D culture, spheroid volume, and number of MDTs with cfDNA concentration were evaluated using Pearson or Spearman correlation tests for normal or non‐normal datasets, respectively. To calculate the IC‐50 of staurosporine, non‐linear regression fit with forced top and bottom values was used. Unless otherwise specified, all the experiments were performed in triplicate.

## 3 Results

### 3.1 cfDNA is a Biomarker of Tumor Burden and Cell Death in Monolayer Culture

Media cfDNA concentrations from cells in 2D culture were first examined to determine the correlation between cfDNA levels with cell number (Figure 2). As shown in Figure 2D,E, cfDNA concentration increased with the number of cells (SK‐LMS‐1: r‐Pearson = 0.96, *p*‐value = 0.0028; MCA‐205: r‐Pearson = 0.95, *p*‐value = 0.0039). Although the cells were untreated, the percentage of dead cells in the wells increased with seeding density, which was potentially due to cell turnover (Figure S3A,B). cfDNA concentration also correlated with PI^+^ area, but to a lesser extent than its correlation with seeding density (SK‐LMS‐1: r‐Pearson = 0.5, *p*‐value = 0.035; MCA‐205: r‐Pearson = 0.77, *p*‐value = 0.0002) (Figure S3C,D), suggesting that both living and dead cells contribute to baseline media cfDNA levels.

**FIGURE 2:**
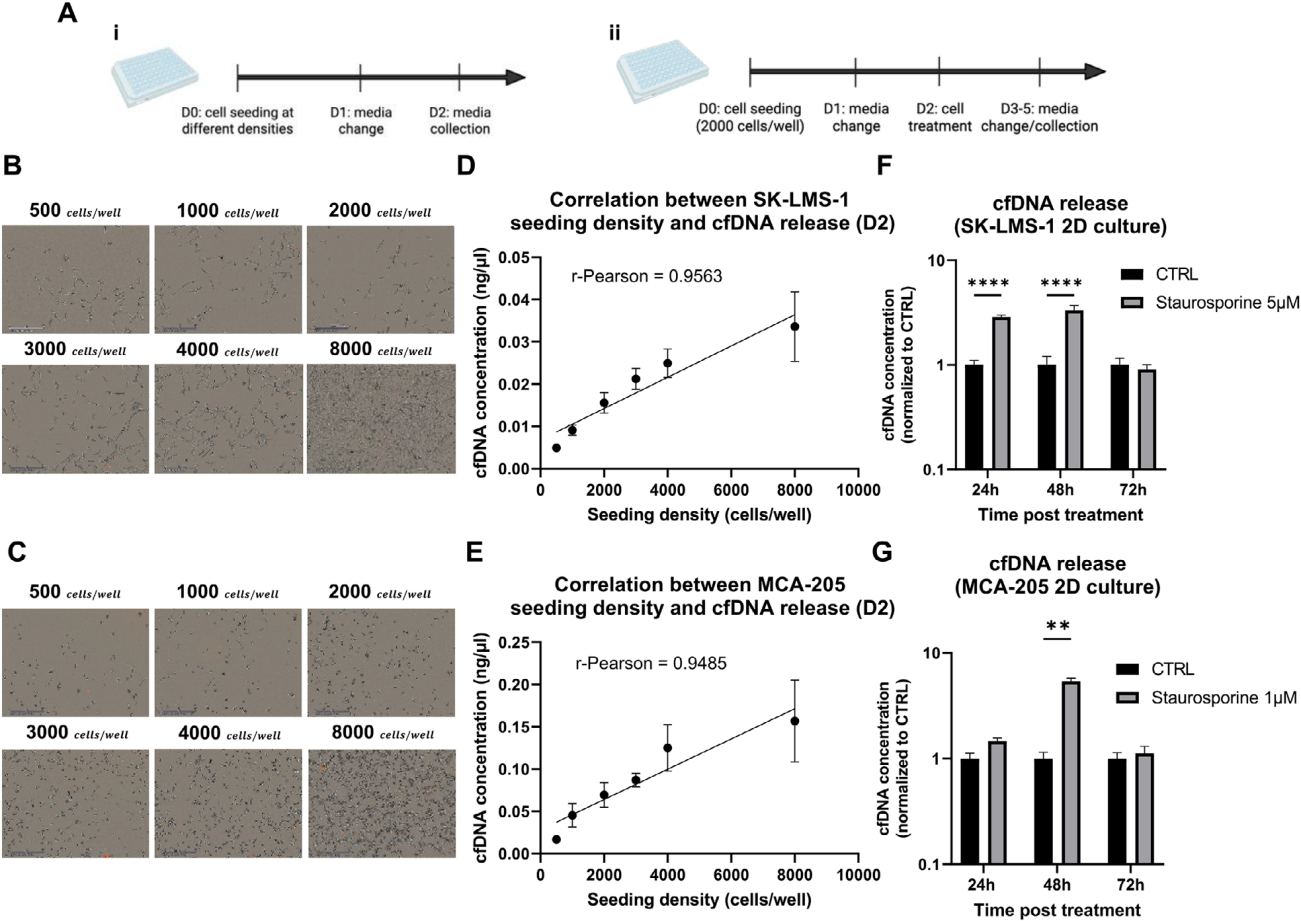
cfDNA concentration in 2D culture correlates with cell number and increases upon apoptotic cell death. SK‐LMS‐1 and MCA‐205 cells were seeded at different densities in a 96‐well plate. (A) The experimental timeline for (i) control cells seeded at different densities and (ii) cells treated with staurosporine. Created using BioRender.com. (B) SK‐LMS‐1 and (C) MCA‐205 2D culture phase‐contrast and immunofluorescence images on day 2. Red objects are Propidium Iodide‐positive (PI^+^) cells. Scale bar = 400 µm. (D,E) The correlation between seeding density and cfDNA concentration in the collected media on day 2 from (D) SK‐LMS‐1 (r‐Pearson = 0.96, *p*‐value = 0.0028) and (E) MCA‐205 (r‐Pearson = 0.95, *p*‐value = 0.0039) cells. Data are represented as mean ± SEM of each experiment (*N* = 3 repetitions per condition). The solid line represents the linear regression fit for each data set. (F) cfDNA concentration over time in media collected from SK‐LMS‐1 cells seeded at 2000 cells/well and treated with 5 µM staurosporine. (G) cfDNA concentration over time in media collected from MCA‐205 cells seeded at 2000 cells/well and treated with 1 µM staurosporine. Data are represented as mean ± SEM of each experiment, normalized to the control level on each day (*N* = 3 repetitions per condition). Statistical analysis was done using two‐way ANOVA with a Šídák's post hoc test for multiple comparisons. ^**^ *p*‐value < 0.01 and ^****^ *p*‐value < 0.0001.

Next, we sought to reproduce previous observations of cfDNA release secondary to staurosporine treatments in 2D STS models [29]. Onset of apoptosis in staurosporine‐treated SK‐LMS‐1 and MCA‐205 cells seeded at 2000 cells/well was confirmed with an increased CC3 level, starting at 3‐6 h post‐treatment and plateauing within 12 h in both cell lines (Figure S4), consistent with other reports [35, 36, 37]. A subsequent peak in the release of cfDNA was observed within 48 h after treatment (Figure 2F,G), also consistent with previous studies [29]. In SK‐LMS‐1 and MCA‐205 cells, staurosporine caused 3.3 and 5.4 times more release of cfDNA in the media 48 h after treatment, respectively. This demonstrates that treatment‐induced apoptosis significantly augments cfDNA release, elevating it well above the baseline levels observed in proliferating cultures.

### 3.2 On‐Chip cfDNA Concentration is a Biomarker of Spheroid Tumor Burden

We hypothesized that larger tumor burden in 3D models cultured in microfluidic chips also correlates with higher media concentrations of cfDNA. SK‐LMS‐1 and MCA‐205 cells were introduced into the chip on day 0 and formed spheroids with increasing sizes depending on the initial seeding density (Figure 3A–C). The crude amount of media cfDNA over time, quantified by qPCR, demonstrated a direct correlation between spheroid size and cfDNA level (Figure 3D,E). Day 3 average spheroid volume (extrapolated from spheroids’ diameter measured from brightfield images assuming a spherical geometry) strongly correlated with cfDNA media level (SK‐LMS‐1: r‐Spearman = 0.76, *p*‐value = 0.0003; MCA‐205: r‐Spearman = 0.73, *p*‐value < 0.0001, Figure 3F,G). This correlation between spheroid volume and cfDNA weakened over culture time (D4‐6) as the spheroids adjusted to their environment (Figure S5). Of note, although MCA‐205 spheroids with the highest seeding density (4 × 10^6^ cells/well) had comparable size to spheroids formed from lower seeding density (2×10^6^ cells/mL), they released considerably more cfDNA, which continued even past day 3. In this specific case, an increase in cfDNA levels beyond the expected baseline served as an indicator of potentially suboptimal culture conditions. This observation was corroborated by the formation of smaller‐than‐anticipated spheroids and evident cell shedding observed under brightfield microscopy (Figure 3A). Consequently, cfDNA levels could assist in identifying tumor burden as well as suboptimal spheroid formation during the experimental optimization process, serving as an early indicator of compromised culture conditions.

**FIGURE 3:**
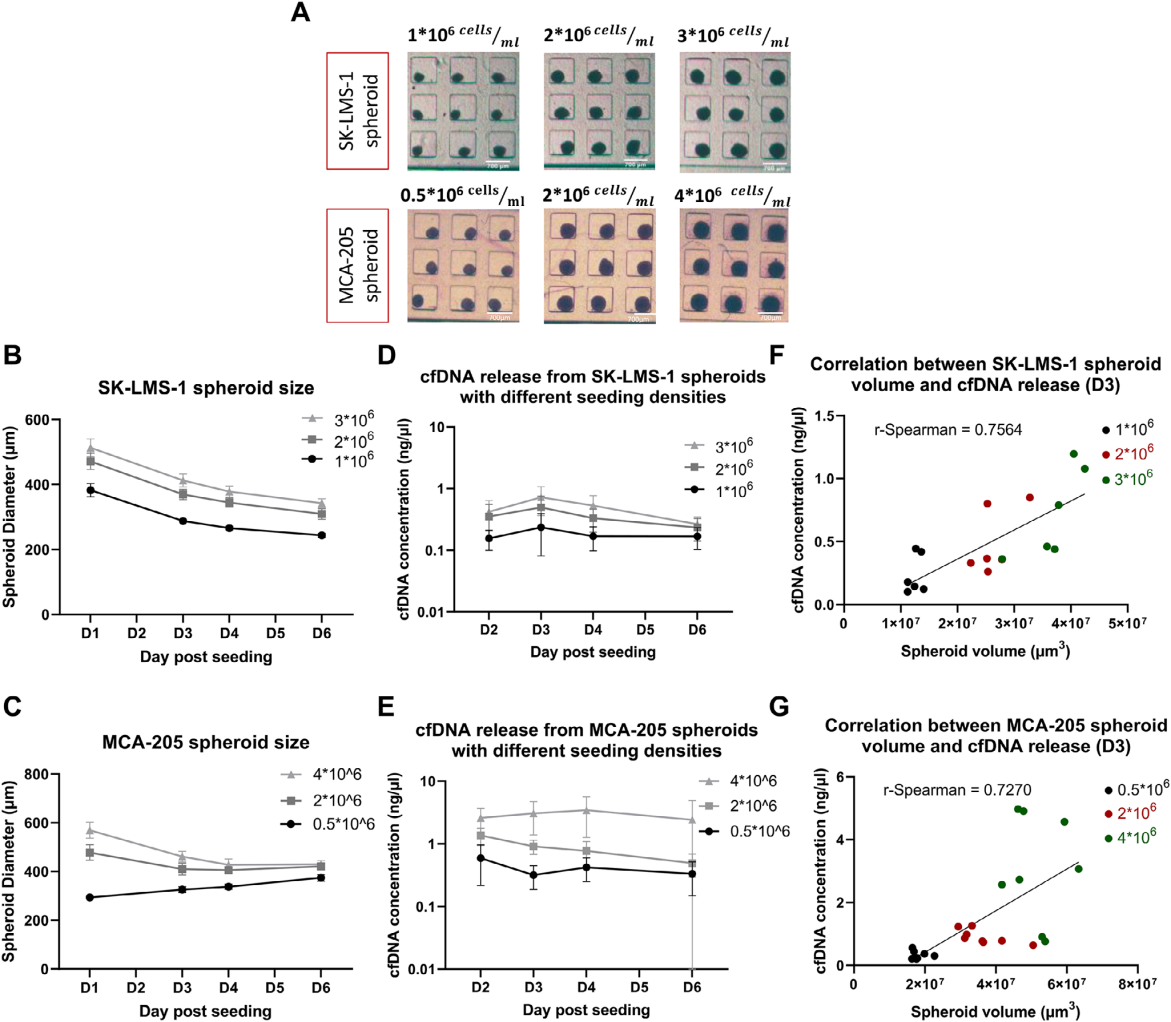
cfDNA concentration in the supernatant of the chip correlates with tumor burden of spheroids. SK‐LMS‐1 and MCA‐205 spheroids were seeded at different densities to obtain spheroids with different sizes. (A) Brightfield images of the spheroids 3 days post‐seeding. Scale bar = 700 µm. (B) SK‐LMS‐1 and (C) MCA‐205 mean spheroid diameter over time (D1‐D6 post‐seeding). Data are represented as mean ± SD of each channel to *N* ≥ 3 repetitions; *N* = 2 for MCA‐205 spheroids on day 4 with 2 channels per repetition. (D,E) cfDNA concentration in the chip's media containing (D) SK‐LMS‐1 and (E) MCA‐205 spheroids over time (D2‐D6 post‐seeding). Data are represented as mean ± SD for each channel. (*N* ≥ 3 repetitions with 2 channels per repetition). (F,G) Spearman correlation between (F) SK‐LMS‐1 (r‐Spearman = 0.76, *p*‐value = 0.0003) and (G) MCA‐205 (r‐Spearman = 0.73, *p*‐value < 0.0001) average spheroid volume (as derived from their diameter) and their cfDNA release on day 3. Each dot represents an average spheroid volume in one channel with its corresponding cfDNA concentration on day 3. Black, red, and green dots represent channels with lowest, medium, and highest seeding densities.

### 3.3 cfDNA as a Marker of Apoptosis in Spheroids

We then explored whether cfDNA is a biomarker of on‐chip treatment‐induced apoptosis by treating SK‐LMS‐1 and MCA‐205 spheroids with 0–20 µM staurosporine for 24 h (Figure 4A). As is apparent in Figure 4B–E, spheroids showed a dose‐dependent cumulative release of cfDNA into the chip's supernatant. In SK‐LMS‐1 spheroids, staurosporine doses of 2.5 µM and above caused 5–10 times more cfDNA release relative to control over the course of five days (Figure 4D). Fitting a non‐linear curve to this graph, we could find the IC‐50 of staurosporine for SK‐LMS‐1 spheroids to be 1.55 µM (Figure S6A). MCA‐205 spheroids were more sensitive to staurosporine, and 0.5 µM was enough to induce around 20 times more cfDNA release from cells (Figure 4E and Figure S6B, IC‐50 = 0.5 µM).

**FIGURE 4:**
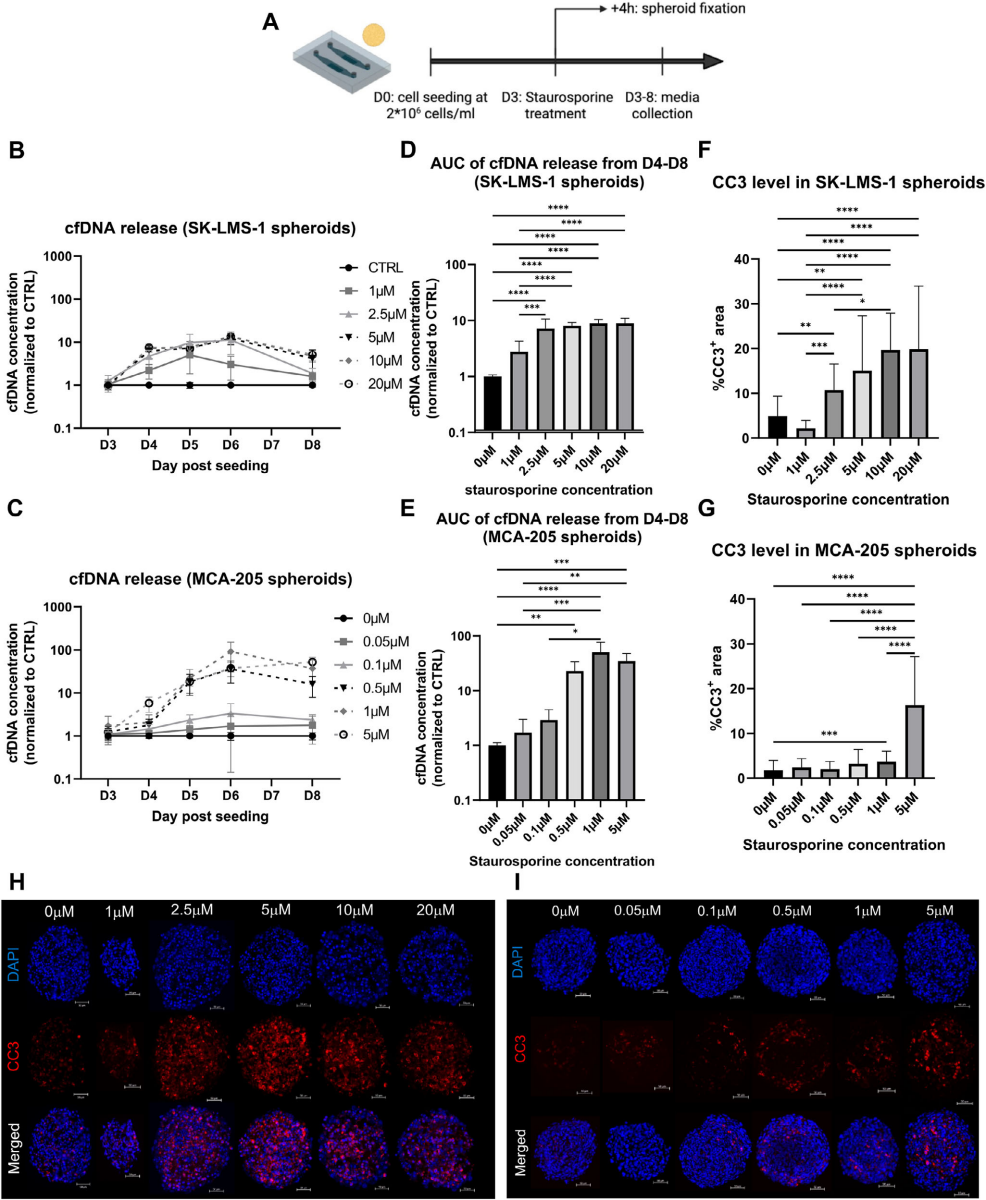
cfDNA is a reliable proxy for apoptosis in spheroids. SK‐LMS‐1 and MCA‐205 spheroids were treated with 0–20 and 0–5 µM of staurosporine, respectively. (A) Experimental timeline for spheroid treatment. Created with BioRender.com. (B,C) cfDNA concentration over time in the chip's media containing (B) SK‐LMS‐1 and (C) MCA‐205 spheroids treated with various doses of staurosporine. Data are represented as mean ± SD of each channel, normalized to the value of control channels for each experiment on each day. (*N* ≥ 3 repetitions with 2 channels per repetition). (D,E) Area under the curve (AUC) of cfDNA release from day 4 to 8 from treated and untreated (D) SK‐LMS‐1 and (E) MCA‐205 spheroids. Data are represented as mean ± SD of each channel, normalized to the value of control channels for each experiment. Statistical analysis was done using one‐way ANOVA with a Tukey post‐hoc test for multiple comparisons. (F,G) Level of CC3 in (F) SK‐LMS‐1 and (G) MCA‐205 spheroids treated with various doses of staurosporine and measured from immunofluorescence staining of fixed spheroids 4 h post‐treatment. Data are represented as mean ± SD of each spheroid. (*N* ≥ 3 repetitions with at least 2 spheroids per repetition). Statistical analysis was done using Kruskal–Wallis with a Dunn post hoc test for multiple comparisons. (G,H) Representative immunofluorescence images of (G) SK‐LMS‐1 and (H) MCA‐205 spheroids stained with DAPI and CC3. Scale bar = 50 µm. ^*^ *p*‐value < 0.05, ^**^ *p*‐value < 0.01, ^***^ *p*‐value < 0.001, and ^****^ *p*‐value < 0.0001.

We observed that for both cell lines, the peak of cfDNA release occurred 3 days post‐treatment, except for MCA‐205 spheroids treated with 5 µM of staurosporine that demonstrated continued cfDNA release peaking 5 days post‐treatment (Figure 4C). This highlights the importance of examining the biomarker over time rather than focusing on a single time point. To support these data, spheroids were fixed 4 h post‐treatment and stained for CC3, which was previously observed to increase within 3–12 h post‐treatment in 2D models. Similar to the trend of cfDNA release in SK‐LMS‐1 spheroids, 2.5–20 µM of staurosporine significantly increased CC3 levels (*p*‐value < 0.01, Figure 4F). However, CnC3 levels at this specific time point were only significantly increased in MCA‐205 spheroids treated with the highest drug concentrations of 1 and 5 µM (Figure 4G). Overall, in our 3D STS models, cfDNA release was delayed and prolonged over several days post‐treatment even though the initiation of apoptosis was observed within hours of staurosporine treatment.

### 3.4 cfDNA as a Biomarker of Tumor Burden and Apoptosis in Ex Vivo Micro‐Dissected Tumor (MDTs) Explants

As spheroids represent one of the simplest forms of 3D culture, we sought to validate cfDNA as a tumor burden and treatment response biomarker in a more complex and physiologically relevant 3D model, such as MDT explants cultured on‐chip. To examine the correlation of tumor burden with media cfDNA levels, an increasing number of SK‐LMS‐1 and MCA‐205 MDTs (2 to 8) were loaded per channel. A larger release of cfDNA during the first days was observed as compared to spheroids, consistent with high stress caused by the MDT production procedure, which involved tissue slicing and punching (Figure 5A,B). On day 1, when most cell death occurred, cfDNA release was strongly correlated with the number of MDTs per channel (Figure 5C,D). SK‐LMS‐1: r‐Pearson = 0.99, *p*‐value = 0.0068; MCA‐205: r‐Pearson = 0.99, *p*‐value = 0.0006). Although cfDNA from SK‐LMS‐1 MDTs still showed clear correlation with the number of MDTs throughout culture (r‐Pearson > 0.89 for day 2, 4, and 6, Figure S7A–C), this correlation progressively weakened for MCA‐205 MDTs, limiting its ability to distinguish channels with different number of MDTs (r‐Pearson (D2) = 0.9, r‐Pearson (D4) = 0.15, r‐Pearson (D6) = −0.19, Figure S7D–F). To confirm that the low level of cfDNA concentration at later time points was not due to the absence of cells in MDTs after the adjustment phase, we stained fixed SK‐LMS‐1 and MCA‐205 MDTs for DAPI and Ki‐67 and confirmed the presence of proliferative cells after 8 days in culture (Figure 5E).

**FIGURE 5:**
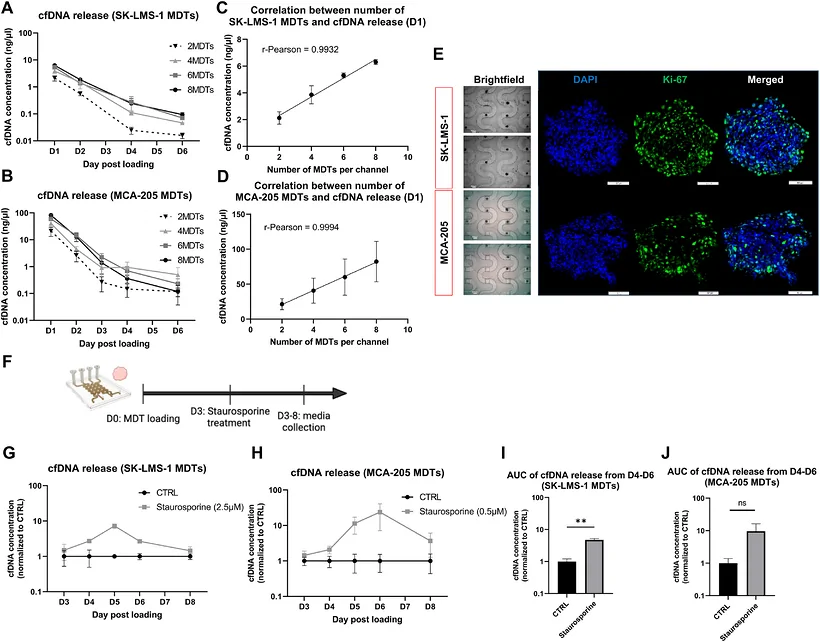
cfDNA concentration in the MDT chip's supernatant correlates with the number of MDTs and increases upon apoptosis. (A–D) cfDNA concentration in the chip's supernatant containing different numbers of (A) SK‐LMS‐1 and (B) MCA‐205 MDTs over time (day 1–day 6 post‐loading) and (C,D) on day 1. Data are represented as mean ± SEM of each experiment. (*N* ≥ 3 repetitions with at least 2 channels per repetition). The correlation was assessed by performing a linear regression and calculating the Pearson correlation coefficient; SK‐LMS‐1: r‐Pearson = 0.99, *p*‐value = 0.0068. MCA‐205: r‐Pearson = 0.99, *p*‐value = 0.0006. (E) Representative brightfield images of SK‐LMS‐1 and MCA‐205 MDTs on‐chip on day 3, along with representative immunofluorescence images of the same MDTs fixed on day 8 and stained for DAPI (nuclei) and Ki‐67 (proliferation marker). Scale bar (brightfield) = 800 µm. Scale bar (immunofluorescence) = 100 µm. (F) Experimental timeline for MDT treatment with staurosporine, created with BioRender.com. SK‐LMS‐1 and MCA‐205 MDTs were treated with 2.5 and 0.5 µM of staurosporine, respectively. (G,H) cfDNA concentration over time in the chip's media containing (G) SK‐LMS‐1 and (H) MCA‐205 MDTs treated with staurosporine. Data are represented as mean ± SEM of each experiment, normalized to the control level on each day (*N* ≥ 3 repetitions with at least 2 channels per repetition). (I,J) AUC of cfDNA release from day 4 to day 6 from staurosporine‐treated (I) SK‐LMS‐1 and (J) MCA‐205 MDTs. Data are represented as mean ± SEM of each experiment normalized to the control level. Statistical analysis was done using a student's *t*‐test. ns = not significant, ^**^ *p*‐value < 0.01.

Next, we treated the MDTs with staurosporine on day 3 (2.5 µM for SK‐LMS‐1 and 0.5 µM for MCA‐205) to induce apoptosis and follow cfDNA dynamics (Figure 5F). In both cell lines, staurosporine caused 4–8 times more cumulative release of cfDNA compared to control over the 3 days post‐treatment period (Figure 5G–J). Our results demonstrated that baseline measures of cfDNA were a surrogate measure of tumor burden in more complex ex vivo 3D models. Subsequently, the cumulative release of cfDNA during the days following apoptosis induction was a dynamic measure of cell death.

### 3.5 Measurement of cfDNA in Non‐Apoptotic Treatment Responses

After validating that cfDNA is a reliable marker of apoptotic cell death in 3D models, we examined its use for measuring other types of cell death, such as necrosis. Using 2D, spheroid, and MDT models, we treated SK‐LMS‐1 cells with H_2_O_2_, a primarily necrosis‐inducing agent at high doses (Figure 6A) [38]. As illustrated in Figure 6B, 5 mM H_2_O_2_ induced approximately 4 times more cfDNA release 24 h after treatment compared to the control. As expected, the peak of cfDNA release occurred 24 h sooner after H_2_O_2_ compared to staurosporine, due to the disruptive nature of necrosis on the cell membrane. Similarly, SK‐LMS‐1 spheroids and MDTs showed a significant and dose‐dependent increase in cfDNA within 24–72 h after H_2_O_2_ treatment (Figure 6C,D and Figure S8, *p*‐values < 0.0001).

**FIGURE 6:**
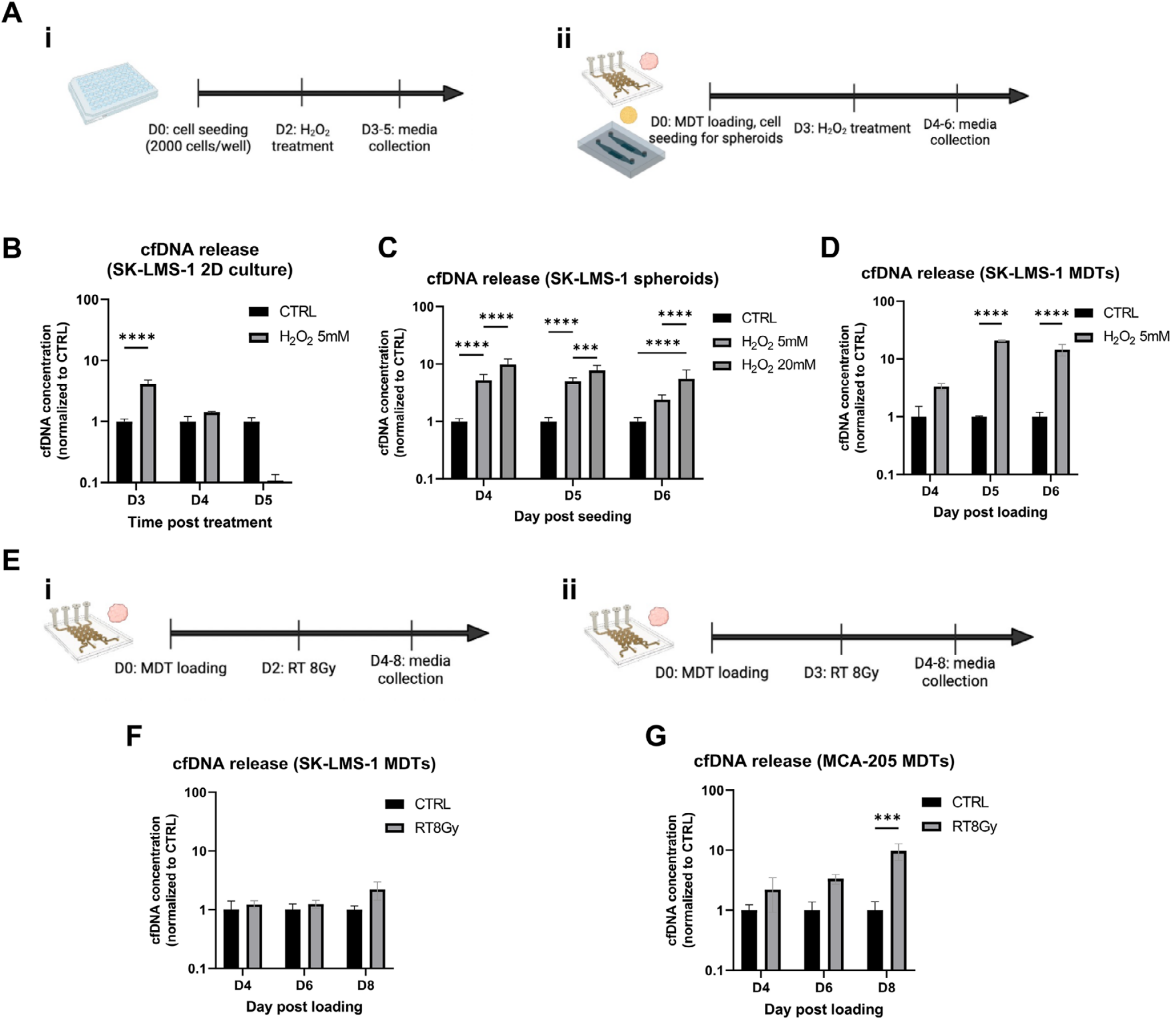
cfDNA dynamics could reflect non‐apoptotic cell death, including necrosis and RT‐induced cell death. (A) Experimental timeline for treatment of SK‐LMS‐1 cells with H_2_O_2_ in (i) 2D and (ii) on‐chip spheroid and MDT culture. Created with BioRender.com. (B) cfDNA concentration over time in media collected from SK‐LMS‐1 2D culture treated with 5 mM H_2_O_2_. Data are represented as mean ± SEM for each experiment, normalized to the control level on each day (*N* = 3 repetitions per condition). (C) cfDNA concentration over time in the chip's media containing SK‐LMS‐1 spheroids treated with 5 and 20 mM of H_2_O_2_. Data are represented as mean ± SD of each channel normalized to the value of control channels for each experiment on each day. (*N* ≥ 3 repetitions with at least 2 channels per repetition). (D) cfDNA concentration over time in the chip's media containing SK‐LMS‐1 MDTs treated with 5 mM H_2_O_2_. Data are represented as mean ± SEM of each experiment, normalized to the control level on each day (*N* = 3 repetitions with at least 2 channels per repetition). (E) Experimental timeline for treatment of (i) SK‐LMS‐1 and (ii) MCA‐205 MDTs with 8 Gy irradiation. Created with BioRender.com. (F,G) cfDNA concentration over time in the chip's media containing (F) SK‐LMS‐1 and (G) MCA‐205 MDTs treated with 8 Gy irradiation. Data are represented as mean ± SEM of each experiment normalized to the control level on each day (*N* ≥ 3 repetitions with at least 2 channels per repetition). Statistical analysis was done using ordinary two‐way ANOVA with Šídák's multiple comparison test. ^***^ *p*‐value < 0.001 and ^****^ *p*‐value < 0.0001.

Finally, we moved forward to test the application of the cfDNA method to a more clinically relevant treatment. As RT is a standard treatment in the management of sarcoma patients, we irradiated SK‐LMS‐1 MDTs with a single 8 Gy dose on day 2 (Figure 6E). The reaction of MDTs to RT was different from that of staurosporine and H_2_O_2_; there was a small and non‐significant increase in cfDNA release, which occurred later in time post‐treatment (6 days after irradiation, Figure 6F). This could be a result of diverse effects that RT has on different tumor cells rather than membrane‐disrupting cell death only. For example, in some cells, RT could induce senescence, that is, a state of durable cell cycle arrest, in which the cell membrane remains intact, and DNA is not released extracellularly [39, 40]. On the other hand, RT could also induce mitotic catastrophe followed by late apoptosis [40]. As an example, MCA‐205 cells showed extensive cell death 5 days after RT in 2D compared to SK‐LMS‐1 cells (Figure S9). To determine whether RT could cause an increase in cfDNA if it induces cell death, we also irradiated MCA‐205 MDTs. Consistent with the greater RT‐induced cell death observed in 2D culture, RT induced a significant increase in cfDNA release in MCA‐205 MDTs 5 days after irradiation (Figure 6G). All in all, increased cfDNA release was observed following all tested treatments (staurosporine, H_2_O_2_, and RT). However, the data suggest that the type of cell death is the critical factor, with cfDNA release being significantly higher from pathways that involve cell membrane disruption.

### 3.6 Association Between the Viability of Patient‐Derived MDT Explants With cfDNA

Having established cfDNA as a robust proxy for cell death in preclinical models, the final step was to validate this method in human patient‐derived sarcoma MDTs. Unlike commercial cell lines, the viability of human MDTs can vary in culture from patient to patient, representing a significant challenge in ex vivo studies. We compared the cfDNA concentration in the chip's media from MDTs from 4 different sarcoma patients, with H&E‐stained MDTs that were fixed at different time points during their culture on chip. As illustrated in Figure 7, the MDTs that ultimately did not survive (MDTs from patients 1 and 2), defined as MDTs with little to no cells seen in H&E after day 4 (Figure 7E,F), released a considerable and increasing amount of cfDNA in the first days, followed by a decline to near zero (Figure 7A,B). On the other hand, MDTs that survived for at least 8 days showed less cfDNA release during the first 5 days (patients 3 and 4 MDTs, Figure 7C,D). Notably, in patient 4 MDTs, a low amount of cfDNA was continuously released, with periodic rises and falls, which may be secondary to longitudinal cell deaths that resulted in the reduction of nuclear area (Figure 7D–H). Collectively, these results provide a proof of concept that cfDNA release patterns can help establish a potential viability threshold for patient‐derived MDTs, although more samples are needed to validate this threshold for different subtypes of sarcoma.

**FIGURE 7:**
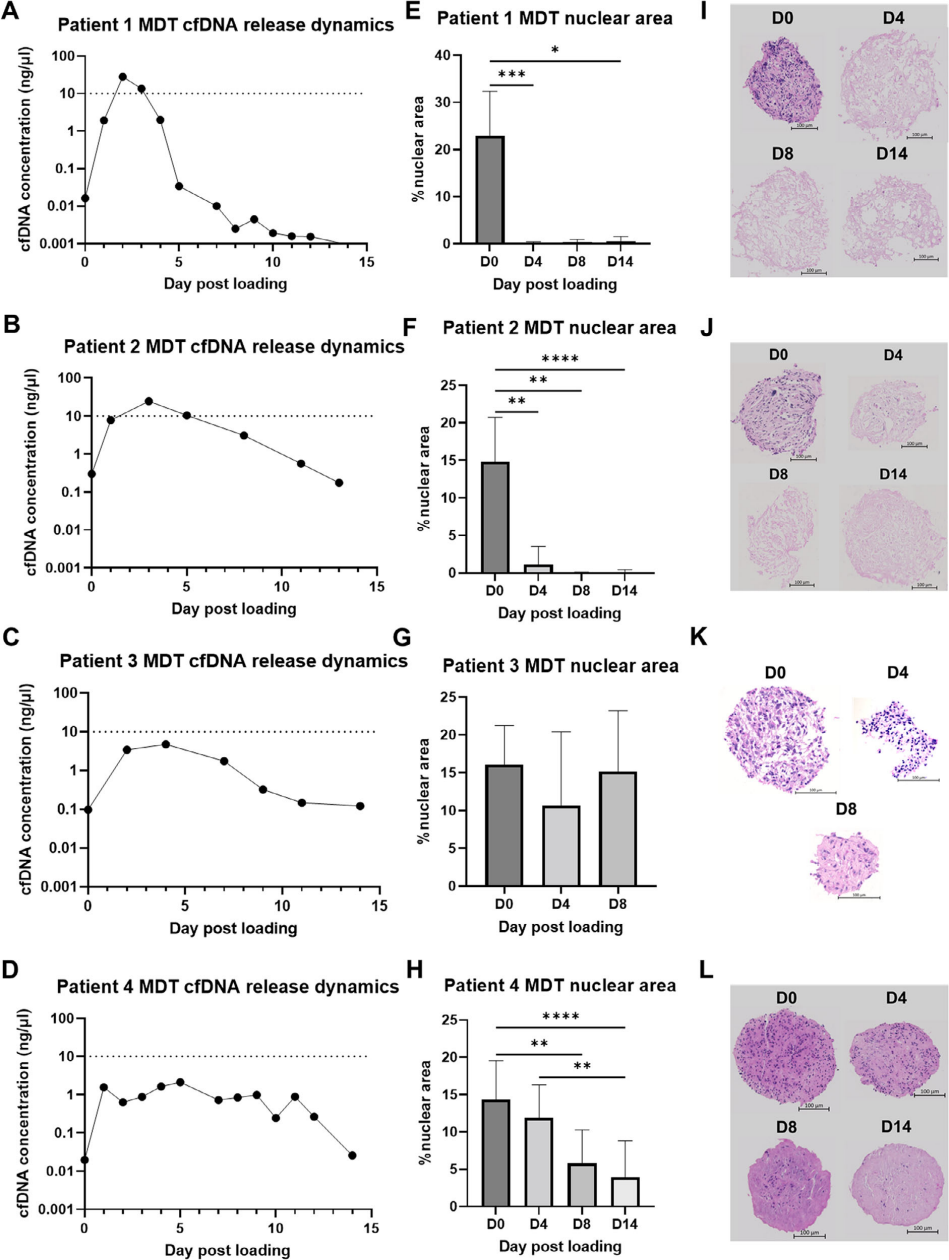
cfDNA is a potential indicator of viability for patient MDTs on‐chip. (A–D) cfDNA concentration in the chip's media containing 4 different sarcoma patient MDTs over time (Day 1–14). For patient 1 and 4 MDTs, the media was changed and collected every 24 h, while for the other two, media change was done every 48–72 h (time points with cfDNA data available). The dashed line is a hypothetical threshold above which the cfDNA release could indicate extensive cell death and loss of viability within sarcoma ex vivo patient MDTs. (E–H) Calculated nuclear area in H&E staining of the same MDTs as in (A–D) fixed on day 0, 4, 8, and 14 after loading. Data are represented as mean ± SD of each MDT. (I–L) Representative H&E images of the same MDTs in (A–D) fixed at different time points. Scale bar = 100 µm. Statistical analysis was done using Kruskal–Wallis with a Dunn post hoc test for multiple comparisons. ^*^ *p*‐value < 0.05, ^**^ *p*‐value < 0.01, ^***^ *p*‐value < 0.001, and ^****^ *p*‐value < 0.0001.

## 4 Discussion

With the increasing adoption of 3D in vitro and ex vivo tumor‐on‐chip models, novel assays need to be developed to assess their viability and cell death in response to cancer treatments. Using sarcoma spheroids and MDTs on‐chip, we showcased the potential of cfDNA measurements as a proxy for cell death in 3D models. We first validated that cfDNA concentration reflected tumor burden (i.e., number of cells) and cell death in 2D culture. Then, by producing spheroids with varied sizes or loading the chips with different numbers of MDTs per channel, we demonstrated that there was a correlation between tumor burden and cfDNA release in the chip's media. This correlation was stronger in the first days of culture and weakened over time as MDTs and spheroids adjusted to their new environment and dead cells and debris were rinsed off with daily media changes. We also established the correlation between treatment‐induced cell death and cfDNA release. By measuring the AUC of cfDNA release, the IC‐50 for staurosporine for each of the cell lines was calculated and was akin to reported values in 3D culture [41, 42]. Finally, cfDNA release was utilized to assess the viability of STS patient MDTs inside the chip. We showed that a significant and increasing amount of cfDNA release in the first days of culture, followed by a rapid decline, could indicate that the chip's condition was not optimal for the MDT culture (e.g., patient 1 and 2 MDTs). It should be noted that the threshold for the maximum cfDNA release beyond which the MDTs are considered not surviving could vary depending on cancer type and should be determined experimentally.

In this study, we emphasized following the cfDNA release dynamics over time. We hypothesize that cfDNA transport inside 3D tumor models depends on a variety of factors, including the mechanism of cell death induced by each treatment and how it affects the tumor's structural integrity. Here, we showed that cfDNA release dynamics were different after treating 3D models with staurosporine, high‐dose H_2_O_2_, and single‐dose RT, with peaks at 48–72 h, 24–48 h, and beyond 120 h post‐treatment. Staurosporine is a widely used drug to induce apoptosis, while high‐dose H_2_O_2_ has been reported to mostly induce necrosis [36, 37, 38]. Still, both drugs have been reported to induce other types of cell death as well, for which extensive mechanistic validation and discrimination were deemed beyond the scope of this paper [43, 44]. Nevertheless, as shown in brightfield images of treated spheroids in Figure S8, H_2_O_2_ disrupted spheroid structure sooner than staurosporine, in accordance with its mostly necrosis‐inducing nature, which resulted in an earlier cfDNA peak. On the other hand, RT, which could induce a variety of effects, including senescence or mitotic catastrophe followed by late apoptosis, led to delayed cfDNA release. This release was far less significant in SK‐LMS‐1 MDTs compared to MCA‐205 MDTs, as expected from 2D culture experiments which showed less cell death after RT for SK‐LMS‐1 cells. In addition to radio‐resistance or limited RT‐induced cell death, the low‐level cfDNA increase in SK‐LMS‐1 cells could have been a result of RT‐induced senescence [40]. Rostami et al. investigated RT‐induced cfDNA release and showed that cfDNA levels increased 72–94 h after RT in some irradiated cell lines. In contrast, other cell lines exhibiting senescence‐like features, such as increased *β*‐galactosidase activity following RT, showed no change or even a decrease in cfDNA concentration [29]. Thus, although our method can determine whether cell death is part of the tumor response, careful consideration is required when interpreting cfDNA release dynamics in response to treatments with complex mechanisms of action, such as RT, as measuring the crude amount of cfDNA cannot definitively discriminate among these effects.

Previous literature studied cfDNA released from well‐based 3D culture; however, not as a means for cell death measurement [45, 46]. The cfDNA method developed here is generally applicable to all 3D models, including in vitro and ex vivo models in various culture systems. In this paper, we focused on on‐chip 3D models as there is an unmet need to develop cell death assays without sacrificing the biospecimens. Moreover, the highly concentrated released cfDNA due to smaller media volume in the chip and the availability of culture media at each media change interval make this method more suited for on‐chip 3D cultures.

Measuring cfDNA concentration in the chip's supernatant as a proxy of cell death has numerous advantages compared to other current methods. First, compared to the methods that require fixing or dissociating the 3D models, cfDNA is quantitative, non‐disruptive, and rapidly quantifiable. Unlike static methods, which necessitate extensive preliminary experiments to identify an optimal time point, continuous cfDNA monitoring captures the complete dynamics of a therapeutic response. This dynamic aspect may explain the incomplete correlation we observed between cfDNA levels and CC3 staining, as the peak of CC3 activation may have occurred after our 4‐hour fixation point, a temporal detail that snapshot endpoint analysis would miss but cfDNA profiling would capture. This is particularly crucial when working with limited patient‐derived samples, where sacrificing tissue for multiple timepoint analysis is not feasible. From a practical standpoint, cfDNA analysis is rapid and cost‐effective, avoiding the use of expensive reagents such as antibodies. Furthermore, unlike methods requiring integrated biosensors, our approach is platform‐agnostic and can be applied to any microfluidic chip, ultra‐low attachment plate, or other 3D culture system without the need for sophisticated microscopy instrumentation.

ctDNA in a patient's blood or other bodily fluids highly reflects the patient's tumor burden [28, 31, 47]. Furthermore, several clinical studies suggested that a rapid increase in ctDNA after treatments followed by a sharp decline to undetectable or below pre‐treatment level is associated with improved prognosis [48, 49]. In this study, we investigated both aspects in 3D models‐on‐chip and observed similar trends. For instance, cfDNA release did not fully correlate with spheroid volume or MDT number at later time points, consistent with clinical data demonstrating that ctDNA levels reflect overall tumor burden, which is associated not only with tumor size, but also with various other tumor traits, including necrotic rate, proliferation, and so on [31, 47]. We propose that the non‐destructive nature of this “on‐chip liquid biopsy” is a significant advantage, enabling longitudinal monitoring of treatment responses in precious patient explants without sacrificing the specimen. This capability maximizes the data obtained from a single sample and holds considerable promise for personalizing therapeutic strategies.

Despite its potential, the current cfDNA methodologies have limitations. A primary challenge is the lack of a standardized method for data normalization. As demonstrated in our study, baseline cfDNA levels correlate with the initial cell number. In addition, when working with highly heterogeneous in vivo tumors, potential necrotic/apoptotic/hypoxic areas within the initial tumor, as well as variable stress‐induced cell death during production of the ex vivo 3D model, will affect the cfDNA release dynamics. This would complicate the interpretation of the cfDNA results. Therefore, to accurately compare treatment‐induced cell death across more complex models, normalization is critical. Current strategies are suboptimal, as they either require disrupting the model for cell counting, which negates the non‐destructive advantage, or rely on viability assays that are not validated for on‐chip 3D cultures. While waiting for robust normalization techniques to be developed, the issue of sample inhomogeneity can be partially mitigated by increasing the number of biological replicates. Previous tissue microarray studies have suggested that three to four 0.6–2 mm diameter biopsy cores could reliably represent tissue heterogeneity [50, 51]. The surface area of three average biopsy cores (1.3 mm in diameter) is equivalent to that of ∼ 20 MDTs measuring 500 µm in diameter. Consistent with this estimate, previous work demonstrated that 15 MDTs were sufficient to recapitulate the histopathological features of the original tumor [11]. As MDT loss during media change is minimal, non‐disruptive media collection from one chip (4*8 MDTs) should be a faithful representation of a tumor. Nevertheless, increasing the biological replicates would further minimize the variability of the 3D models generated from a heterogeneous tumor. A second limitation is the inability to distinguish between cfDNA released from different types of cell death (apoptosis, necrosis, etc.) as well as cfDNA that is passively released from dying cells and which is actively secreted by viable cells. A therapeutic agent that stimulates active cfDNA secretion, for example through extracellular vesicles, without inducing cytotoxicity could be misinterpreted as a cell‐killing agent [52]. Finally, quantifying total cfDNA cannot distinguish its cellular origin in co‐culture models. Future work could overcome this by employing more advanced techniques, such as whole‐genome or targeted sequencing, to identify cell‐type‐specific genetic or epigenetic markers within the cfDNA.

## 5 Conclusions

In conclusion, this study demonstrated the use of cfDNA measurement in the chip's media as a reliable and non‐destructive proxy for assessing cell death in on‐chip 3D tumor models. This cost‐effective and rapid assay provides valuable insight into the temporal response of tumor models to various treatments, including cytotoxic agents and radiotherapy, without the need for sophisticated microscopy instrumentation, label‐based detection, or genetic modification. The observed correlations between cfDNA release and tumor cell death in patient explants have the potential to be used to guide personalized treatment strategies. Future work evaluating the sequence of cfDNA fragments could further enhance the technique's accuracy and capability to estimate treatment therapeutic windows.

## Author Contributions

M.Z., P.W., J.L., and T.G. conceptualized the study. M.Z. and J.L. developed the methodology; M.Z. synthesized the experimental data for spheroids and xenografted MDTs. K.K. and B.I.S. synthesized the experimental data for patient MDTs. M.Z. analyzed and interpreted all the data. P.W., T.G., and K.T. provided resources. M.Z. prepared the original draft of the paper. M.Z. J.L., P.W., and T.G. reviewed and edited the draft. P.W., T.G., K.T., and F.R. supervised the project and helped interpret the data. All authors have read and agreed to the published version of the manuscript.

## Funding

This research was funded by the New Frontiers in Research Fund (grant# NFRFR‐2021‐00294), the Cancer Research Society (Grant #1058362), the Panov Program in Precision Chemotherapy at Mount Sinai Hospital, Tier 2 Canada Research Chair, and the Natural Sciences and Engineering Research Council of Canada (grant# RGPIN‐2020‐06838). MZ was supported by TransMedTech Institute, Fonds de recherche du Québec (DOI: https://doi.org/10.69777/328040), and Bourse Canderel.

## Ethics Approval

All patients whose tumor specimens were used in this study provided written informed consent under institutional research ethics board (REB) protocols at Mount Sinai Hospital in Toronto, Canada (01‐0138‐U).

## Conflicts of Interest

Philip Wong and Thomas Gervais are co‐founders and shareholders of MISO Chip Inc., the company from which the microfluidic explant culture devices were purchased. MISO Chip Inc. did not provide funding for this project and had no role in study design, data collection, data analysis, manuscript preparation, or publication decisions. All other authors declare they have no competing interests.

## Supporting information




**Supporting File**: adhm71792‐sup‐0001‐SuppMat.docx.

## Data Availability

The datasets generated and/or analysed during the current study are available in the Borealis repository, https://doi.org/10.5683/SP3/ZUGBRF.
