# Supplementary material for: Cell‐Free DNA as a Biomarker of Cell Death and Treatment Response for On‐Chip Three‐Dimensional Tumor Models
Source: Adv Healthc Mater. 2026 Oct 4;15(40):e71792. doi: 10.1002/adhm.71792 (PMC13712200; doi:10.1002/adhm.71792)
Supplement: Supplementary file 1 [file ADHM-15-0-s001.docx]

Supplementary Materials of

**Cell Free DNA as a Biomarker of Cell Death and Treatment Response for On-Chip Three-Dimensional Tumor Models**

Maryam Ziaee^1,2,3^, Julie Lafontaine^2,3^, Badr Id Said^4,5^, Kousha Kamal^6^, Kim Tsoi^7^, Francis Rodier^2,3,8^, Thomas Gervais^2,3,9^, Philip Wong^2,4,5 *^

*Corresponding author’s Email: [Philip.wong4@uhn.ca](mailto:Philip.wong4@uhn.ca)

Email: [maryam.ziaee@etud.polymtl.ca](mailto:maryam.ziaee@etud.polymtl.ca), thomas.gervais@polymtl.ca

**Table S1.** Histopathology information of the sarcoma patient tumors.

|  | **Age** | **Sex** | **Grade (FNCLCC)** | **Histology** | **Location** |
| --- | --- | --- | --- | --- | --- |
| **Patient 1** | 60 | M | 2 | Undifferentiated pleomorphic sarcoma | Thigh |
| **Patient 2** | 63 | M | 3 | Undifferentiated spindle cell sarcoma | Calf |
| **Patient 3** | 66 | M | 2 | Myxofibrosarcoma | Upper arm |
| **Patient 4** | 49 | M | 2 | Leiomyosarcoma | Thigh |

**Table S2.** Forward and reverse primers’ sequence for qPCR amplification of human and mouse long interspersed nuclear element (LINE) regions.

|  | **Forward primer** | **Reverse primer** | **Amplicon size** | **Source** |
| --- | --- | --- | --- | --- |
| **Human** | 5′-TCACTCAAAGCCGCTCAACTAC-3′ | 5′-TCTGCCTTCATTTCGTTATGTACC-3′ | 82 bp | (Rostami et al. 2020) |
| **Mouse** | 5′-GCCAGGTATCTGTGCATCTT-3′ | 5′-ACTCTAGCTCTCTCCTGAGTTT-3′ | 99 bp | Alekseeva et al. 2020) |

***Figure S1.*** *The schematic and characteristics of the microfluidic chip used for (A) formation and manipulation of spheroids and (B) culturing micro-dissected tissue explants (MDTs).*

***Figure S2. (A&B)*** *Representative amplification curves of standard genomic DNA amplified using primers against long interspersed nuclear elements (LINE) from* ***(A)*** *human and* ***(B)*** *mouse origin.* ***(C&D)*** *Standard curves for* ***(C)*** *human (efficiency = 96%, slope = -3.41) and* ***(D)*** *mouse (efficiency = 97%, slope = -3.4) DNA built using the CT values of the serial dilution of genomic DNA shown in A and B.*

***Figure S3.*** *(A&B) Correlation between Propidium Iodide positive (PI^+^) area and seeding density in (A) SK-LMS-1 (r-Pearson = 0.96, p-value = 0.0028) and (B) MCA-205 (r-Pearson = 0.95, p-value = 0.003) cells seeded in 96 well plates at 500-8000 densities. Data represented as mean*$\pm$*SEM (N =3 repetitions per condition). (C&D) Correlation between cfDNA concentration and PI^+^ area in (C) SK-LMS-1(r-Pearson = 0.5, p-value = 0.0350) and (D) MCA-205 cells (r-Pearson = 0.77, p-value = 0.0002) seeded in 96 well plates at 500-8000 seeding densities. Each dot represents average PI^+^ area in one experiment with its corresponding average cfDNA concentration. The solid line represents the linear regression fit for each data set. The correlations were assessed by calculating Pearson correlation coefficient. (N = 3 repetitions per condition)*

***Figure S4.*** *(A&B) Phase-contrast and immunofluorescence images of (A) SK-LMS-1 and (B) MCA-205 cells cultured in 96 well plates. Cells were treated with Staurosporine at 0 h. Green objects are dead cells stained with CellEvent™ Caspase-3/7 Green ReadyProbes™. Scale bar = 400 µm. (C&D) CC3^+^ area in (C) SK-LMS-1 and (D) MCA-205 cells over 24 h period after Staurosporine treatment. The Green object area was calculated in images as in A&B. (N=1 repetition per condition).*

***Figure S5.*** *(A&B) Spearman correlation between SK-LMS-1 average spheroid volume and their cfDNA release on (A) Day 4 and (B) Day 6. D4: r-Spearman = 0.8, p-value < 0.0001. D6: r-Spearman = 0.44, p-value = 0,.069. (N ≥ 3 repetitions with at least 1 channel per repetition.) (C&D) Spearman correlation between MCA-205 average spheroid volume and their cfDNA release on (C) Day 4 and (D) Day 6. D4: r-Spearman = 0.73, p-value = 0.0087. D6: r-Spearman = 0.7, p-value = 0.0026. (N ≥ 2 repetitions with at least 1 channel per repetition.) Each dot represents average spheroids volume in one channel with its corresponding cfDNA concentration on each day. Black, red, and green dots represent channels with lowest, medium, and highest seeding densities.*

***Figure S6.*** *Area under the curve (AUC) of cfDNA release from Day 4 to Day 8 from (A) SK-LMS-1 and (B) MCA-205 spheroids treated with various doses of staurosporine. Data represented as mean ± SD of each channel normalized to the value of control channels for each experiment. (N* $\geq$ *3 repetitions with at least 2 channels per repetition). Non-linear regression fit was performed to calculate IC-50 for each cell line (SK-LMS-1: IC50 = 1.55 µM; MCA-205 = 0.5µM).*

***Figure S7.*** *(A-C) cfDNA concentration in the chips’ supernatant containing different numbers of SK-LMS-1 MDTs on (A) Day 2, (B) Day 4, and (C) Day 6. The correlation was assessed by performing a linear regression and calculating Pearson correlation coefficient; D2: r-Pearson = 0.9, p-value = 0.11. D4: r-Pearson = 0.91, p-value = 0.09. D6: r-Pearson = 0.99, p-value = 0.0021. (D-F) cfDNA concentration in the chips’ supernatant containing different numbers of MCA-205 MDTs on (D) Day 2, (E) Day 4, and (F) Day 6. The correlation was assessed by performing a linear regression and calculating Pearson correlation coefficient; D2: r-Pearson = 0.9, p-value = 0.11. D4: r-Pearson = 0.15, p-value = 0.85. D6: r-Pearson = -0.19, p-value = 0.81. Data represented as mean ± SEM of each experiment. (N ≥ 3 repetitions with at least 2 channels per repetition).*

***Figure S8.*** *Morphology of spheroids 24 h after H_2_O_2_ and Staurosporine treatment. Scale bar = 700 µm.*

***Figure S9.*** *(A) Phase-contrast and immunofluorescence images of SK-LMS-1 and MCA-205 cells cultured in 96 well plates five days after irradiation. Red objects are dead cells stained with Propidium Iodide (PI). Scale bar = 400 µm. (B) Cell death in SK-LMS-1 and MCA-205 cells 5 days after irradiation. The PI^+^ area was calculated in images as in A and was normalized to the confluency of the cells. Data represented as mean*$\pm$*SEM of each experiment (N = 3 repetitions per condition). Statistical analysis was done using t-student test ***p-value < 0.001.*
